# The Nedd4-binding protein 3 (N4BP3) is crucial for axonal and dendritic branching in developing neurons

**DOI:** 10.1186/1749-8104-8-18

**Published:** 2013-09-17

**Authors:** Michael J Schmeisser, Susanne J Kühl, Michael Schoen, Nathalie H Beth, Tobias M Weis, Andreas M Grabrucker, Michael Kühl, Tobias M Boeckers

**Affiliations:** 1 Institute for Anatomy and Cell Biology, Ulm University, D-89081 Ulm, Germany; 2 Institute for Biochemistry and Molecular Biology, Ulm University, D-89081 Ulm, Germany; 3 WG Molecular Analysis of Synaptopathies, Neurology Department, Neurocenter of Ulm University, Ulm University, D-89081 Ulm, Germany

**Keywords:** Axon, Branching, Dendrite, Nedd4, Neuron, Ubiquitin, *Xenopus laevis*

## Abstract

**Background:**

Circuit formation in the nervous system essentially relies on the proper development of neurons and their processes. In this context, the ubiquitin ligase Nedd4 is a crucial modulator of axonal and dendritic branching.

**Results:**

Herein we characterize the Nedd4-binding protein 3 (N4BP3), a Fezzin family member, during nerve cell development. In developing rat primary hippocampal neurons, endogenous N4BP3 localizes to neuronal processes, including axons and dendrites. Transient *in vitro* knockdown of N4BP3 in hippocampal cultures during neuritogenesis results in impaired branching of axons and dendrites. In line with these findings, *in vivo* knockdown of n4bp3 in *Xenopus laevis* embryos results in severe alteration of cranial nerve branching.

**Conclusions:**

We introduce N4BP3 as a novel molecular element for the correct branching of neurites in developing neurons and propose a central role for an N4BP3-Nedd4 complex in neurite branching and circuit formation.

## Background

Depending on the pattern and extent of how axons and dendrites undergo branching, a neuron has the ability to regulate the level of wiring and the strength of synaptic circuits. In this context, the ubiquitin proteasome system (UPS) has emerged as a decisive regulatory pathway [1-12]. Accumulating data underline the fact that Nedd4 (neural precursor cell expressed and developmentally downregulated), a HECT (homologous to E6-AP carboxyl terminus) domain ubiquitin ligase [13], is of special importance for neuronal network formation because of its capacity to regulate the growth and arborization of both dendrites and axons [14-18]. In the murine brain, for example, Nedd4 turns down Ras-related protein 2A (Rap2A) function, thus inhibiting Rap2 effector kinases of the Traf2- and Nck-interacting kinase (TINK) family and thereby promoting dendritic growth and arborization [16]. Nedd4 was further shown to promote phosphatidylinositol 3-kinase (PI3K)-induced cytoskeletal rearrangements via UPS-mediated downregulation of the Nedd4 substrate phosphatase and tensin homolog (PTEN) to generate a terminally branched arbor in axons of *Xenopus laevis* retinal ganglion cells (RGCs) [15]. The same Nedd4-dependent regulatory mechanism was recently confirmed in axons of dorsal root ganglion (DRG) sensory neurons in the peripheral nervous system [18]. In line with these findings, phrenic nerve fasciculation is severely disrupted in *Nedd4*-null mutants during embryonic development *in vivo*[19]. Interestingly, various Nedd4-interacting proteins serving as accessory and/or adaptor molecules to recruit, sequester or modify Nedd4 have been identified in recent years [20,21]. One of these potential adaptor proteins is the Nedd4-binding protein 3 (N4BP3). It was originally isolated as an interaction partner of Nedd4 in a yeast two-hybrid screen [22]. On the basis of *in vitro* assays, the authors of that study subsequently identified the consensus PY motif (PPxY) in N4BP3 as the binding module for Nedd4, demonstrated that N4BP3 and Nedd4 colocalize in cytoplasmic vesicles and revealed that N4BP3 is not a ubiquitylation substrate of Nedd4.

In our present study, we show that N4BP3 is present in early neurites, growth cones, axons and dendrites of developing primary hippocampal neurons. We subsequently demonstrate that *in vitro* knockdown (KD) of N4BP3 in hippocampal cultures leads to impaired axonal and dendritic branching. We extend these findings *in vivo* by morpholino oligonucleotide (MO)-mediated KD of n4bp3 in developing *X. laevis* embryos, which results in abnormal branching of cranial nerves. On the basis of these findings, we propose that N4BP3 is essential for proper nerve cell development.

## Results

### Nedd4-binding protein 3, a novel Fezzin family member

**Figure 1:**
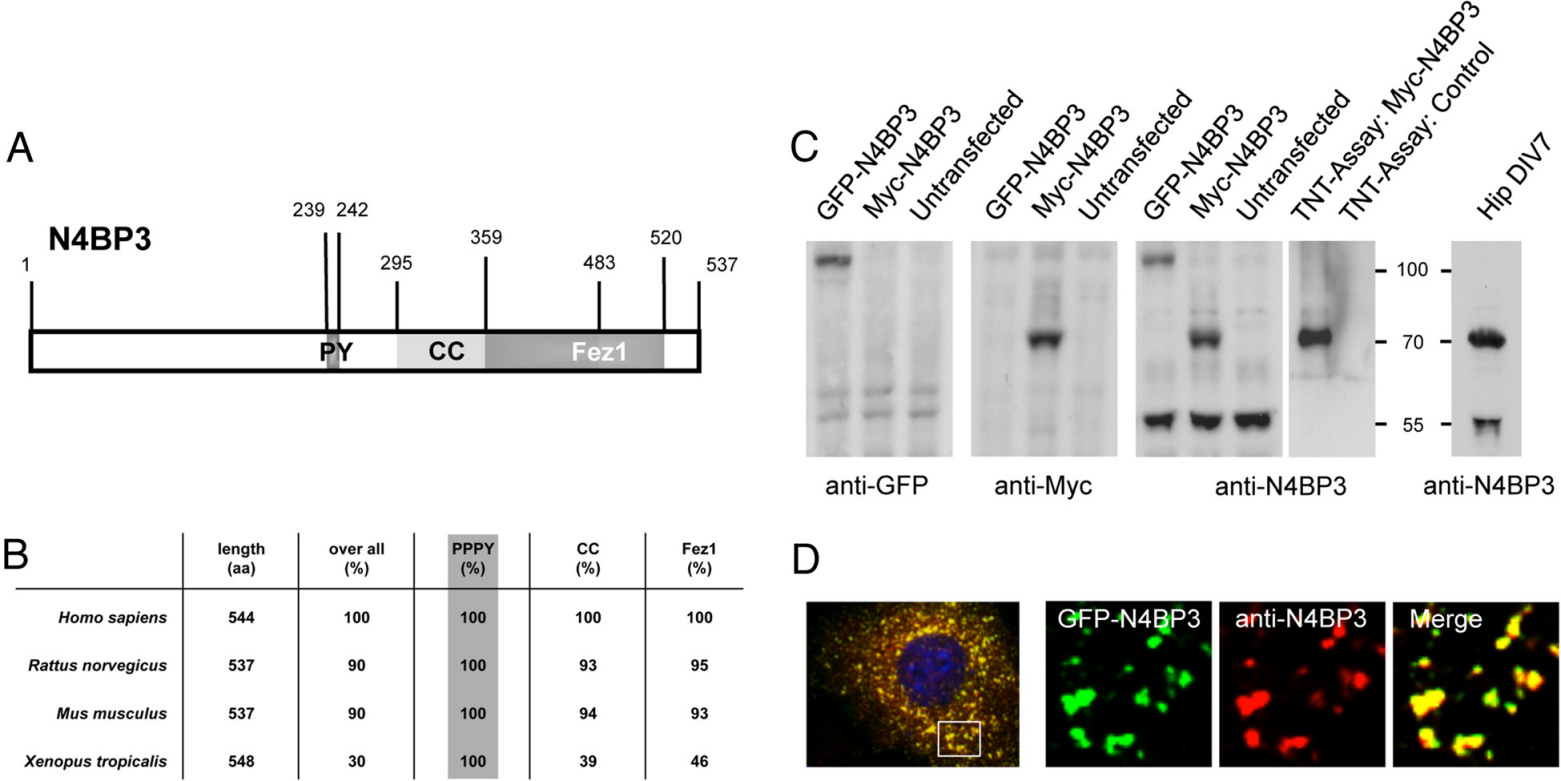
**Analysis of the Nedd4-binding protein 3 domain structure and characterization of an affinity-purified polyclonal Nedd4-binding protein 3 antibody. (A)** Schematic overview of the Nedd4-binding protein 3 (N4BP3) domain structure in *Rattus norvegicus*. The Nedd4 PY-binding domain (aa 239 to 242, PPPY [22]), coiled-coil (CC; aa 295 to 483) and Fez1 (aa 359 to 520) domains are highlighted. Sequence information of the CC and Fez1 domains derives from the Pfam 26.0 database ( http://pfam.sanger.ac.uk/). **(B)** Homology analysis of N4BP3 primary structure (amino acids, full-length protein (overall), PY (PPPY), CC and Fez1 domains). Note the 100% homology within the Nedd4-interacting PY domain between species. **(C)** To characterize the affinity-purified polyclonal N4BP3 antibody, green fluorescent protein (GFP)-N4BP3 and Myc-N4BP3 were expressed in HEK-293T cells. Both fusion proteins were biochemically detected at their predicted molecular weights (70 vs. 100 kDa) in Western blot analysis by either the N4BP3 antibody or GFP and Myc antibodies, respectively. Furthermore, the affinity-purified antibody detects both *in vitro* translated Myc-N4BP3 (transcription/translation (TNT) assay) and endogenous N4BP3 in lysate from DIV7 primary hippocampal cultures at approximately 70 kDa. **(D)** GFP-N4BP3 (Alexa Fluor 488 dye (Molecular Probes/Invitrogen, Eugene, OR, USA), green) is expressed in dotlike structures localized all throughout the cytosol, excluding the nucleus. The same subcellular structures are immunodetected by the N4BP3 antibody (Alexa Fluor 568 dye (Molecular Probes/Invitrogen), red). See insets for clear overlay of green and red channels.

Rat N4BP3 [NCBI:DQ176639] is a protein of 537 amino acids, which, as Fezzin family members ProSAP-interacting protein 1/leucine zipper putative tumor suppressor 3 (ProSAPiP1/Lzts3) [23], PSD-Zip70/leucine zipper putative tumor suppressor 1 (PSD-Zip70/Lzts1) [24] and LAPSER1/leucine zipper putative tumor suppressor 2 (Lzts2) [25], exhibits a central coiled-coil domain and a C-terminal Fez1 domain. In addition, a PY domain (PPxY consensus sequence), which mediates the interaction with the WW domains of Nedd4 [22], can be found exclusively in this Fezzin (Figure 1A). Contrary to ProSAPiP1, PSD-Zip70 and LAPSER1, all of which harbor a classic PDZ domain-binding motif at the very C-terminus, N4BP3 only exhibits a rudimentary one (−SSKI). In line with these structural discrepancies, N4BP3 is not as closely related to the other three Fezzins [23]. However, its PY domain is highly conserved among species (Figure 1B). Western blot analysis with an affinity-purified polyclonal antibody raised against the N-terminus of rat N4BP3 identified both green fluorescent protein (GFP)- and Myc-tagged N4BP3 at their predicted molecular weights (100 vs. 70 kDa), as well as the endogenous protein at approximately 70 kDa in lysate from DIV7 primary hippocampal cultures (Figure 1C). Furthermore, the same antibody was used for immunodetection of GFP-N4BP3 in COS-7 cells (Figure 1D).

### Nedd4-binding protein 3 is enriched in growth cones, axons and dendrites of developing hippocampal neurons

**Figure 2:**
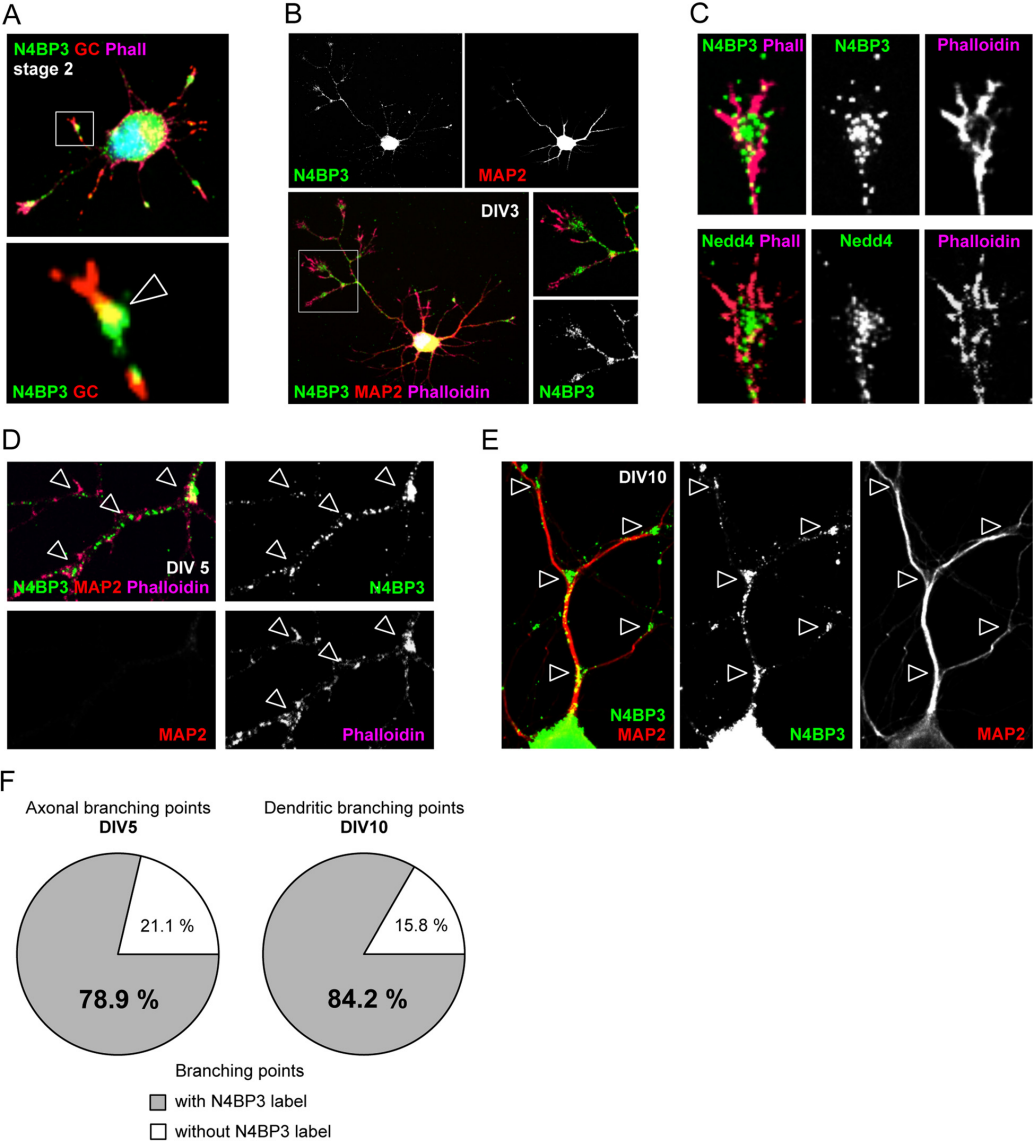
**Spatial localization of Nedd4-binding protein 3 in primary hippocampal neurons.** Immunostaining of Nedd4-binding protein 3 (N4BP3) (Alexa Fluor 488 dye, green) in developing rat primary hippocampal neurons. **(A)** Colocalization of N4BP3 and the axonal growth cone (GC) marker protein 2G13P (Alexa Fluor 568 dye, red) in a stage 2 neuron. Note localization of N4BP3 at the GC base (framed arrowhead). **(B)** In stage 3 neurons at DIV3, N4BP3 is clearly localized within the axon, at axonal branching points and in axonal GCs. **(C)** N4BP3 (Alexa Fluor 488 dye, green; upper row) shows the same spatial distribution as interaction partner Nedd4 (Alexa Fluor 488 dye, green; lower row) within axonal GCs of stage 3 hippocampal neurons at DIV3. **(D)** and **(E)** N4BP3 labeling of both axonal branching points (**D**; framed arrowheads) and dendritic branching points (**E**; framed arrowheads) at DIV5 and DIV10, respectively. **(F)** Pie charts illustrating the percentage of branching points with (gray color) or without (white color) N4BP3 labeling in axons at DIV5 (left) and dendrites at DIV10 (right). **(A)** through **(D)** The actin cytoskeleton is visualized by phalloidin (Alexa Fluor 647 dye, magenta). **(B)**, **(D)** and **(E)** Coimmunostaining with the dendritic marker protein microtubule-associated protein 2 (MAP2; Alexa Fluor 568 dye, red).

In the next set of experiments, rat primary hippocampal cultures were immunostained with the affinity-purified N4BP3 antibody to thoroughly analyze N4BP3 expression and localization throughout neuronal development. N4BP3 is found in early growth cones, as revealed by coimmunostaining of stage 2 neurons with a growth cone marker antibody (arrowhead in Figure 2A). N4BP3 further accumulates in the axon (Figure 2B; insets highlight N4BP3 immunoreactivity in branching points and growth cones, respectively) and shows the same spatial distribution as interaction partner Nedd4 in axonal growth cones at DIV3 (Figure 2C). As branching points of both axons (DIV5, Figure 2D) and developing dendrites (DIV10, Figure 2E) contain N4BP3, we next thoroughly analyzed N4BP3 labeling of branching points and found that N4BP3 labeling was present in 78.9% (±1.9%) of axonal branching points and 84.2% (±2.6%) of dendritic branching points, respectively.

### Knockdown of Nedd4-binding protein 3 in developing primary hippocampal cultures alters axonal and dendritic branching

**Figure 3:**
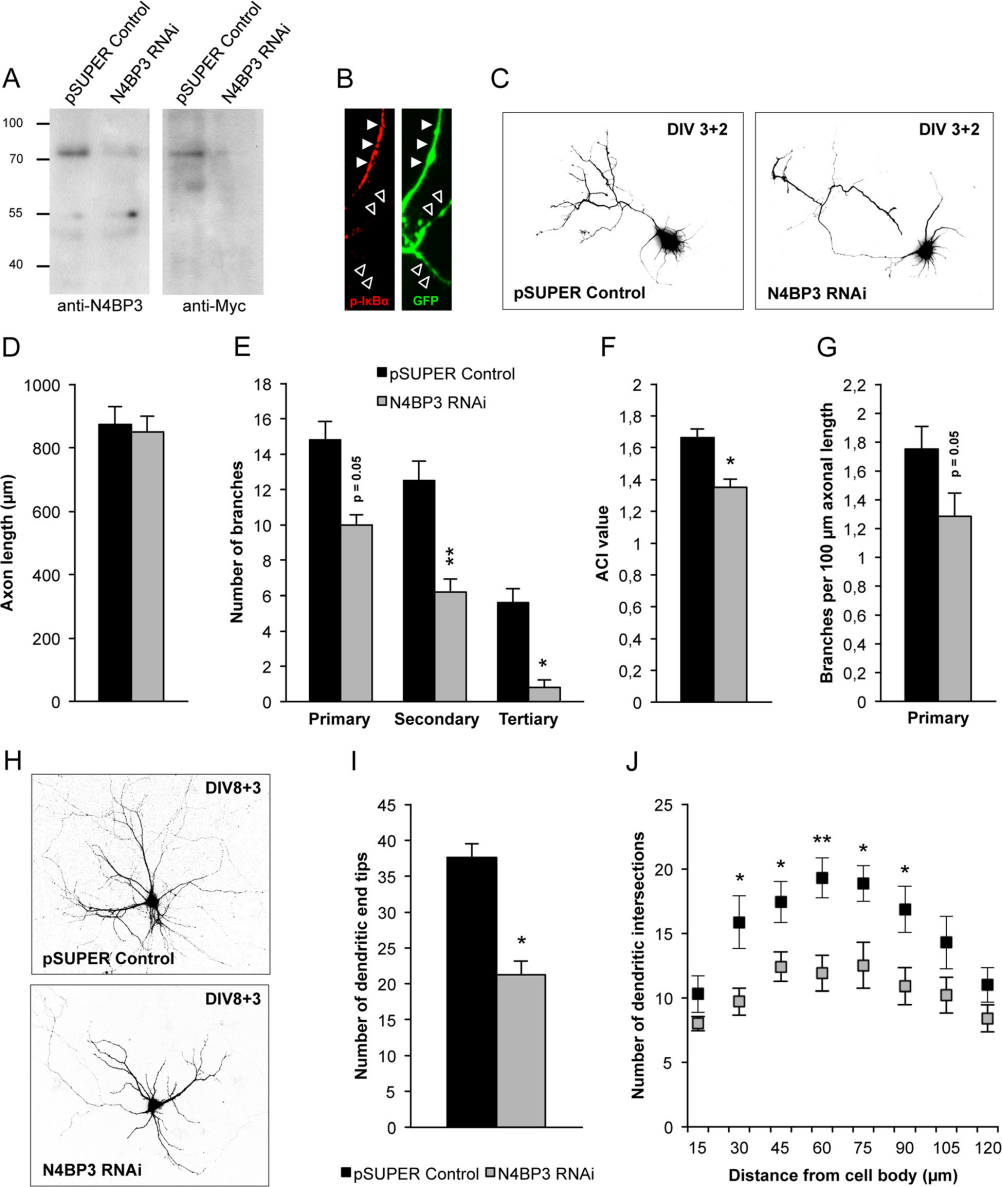
**Transient Nedd4-binding protein 3 knockdown results in impaired neurite branching. (A)** Characterization of the Nedd4-binding protein 3 (N4BP3) interfering RNA (RNAi) construct. HEK-293T cells were cotransfected with Myc-N4BP3 and either the empty pSUPER control vector (Oligoengine, Seattle, WA, USA) or the N4BP3 RNAi construct. Western blot of the corresponding cell lysates shows reduction of Myc-N4BP3 in the presence of N4BP3 RNAi with either N4BP3 or Myc antibodies as indicated. **(B)** All culture wells used for transient transfection experiments were immunostained with a phosphorylated inhibitor of κB, subunit α (p-IκBα) antibody (Alexa Fluor 568, red; left panel) to delineate the axon initial segment (AIS; filled arrowheads) to distinguish between axon (filled arrowheads) and dendrites (framed arrowheads). GFP, green fluorescent protein. **(C)** Representative images of primary rat hippocampal neurons transiently transfected (DIV3 + 2) with either the empty pSUPER control vector or the N4BP3 RNAi construct as indicated. Statistical evaluation of axon length **(D)**; the number of primary, secondary and tertiary branches **(E)**; the axon complexity index (ACI) **(F)**; and the (primary) branches per 100-μm axonal length **(G)** of pSUPER control vector (black bars) vs. N4BP3 RNAi (gray bars) transfected neurons. **(H)** Representative images of rat hippocampal neurons transiently transfected (DIV8 + 3) with either the empty pSUPER control vector or the N4BP3 RNAi construct as indicated. Statistical evaluation of the total number of dendritic end tips **(I)** and Sholl analysis **(J)** of pSUPER control vector (black bar in **(I)**, squares in **(J)**) vs. N4BP3 RNAi (gray bar in **(I)**, gray squares in **(J)**) transfected neurons. *n* = 10 cells from three independent experiments in **(D)**, **(E)**, **(F)**, **(G)**, **(I)** and **(J)** (Student’s *t*-test; *P < 0.05, **P < 0.01, ***P < 0.001).

**Figure 4:**
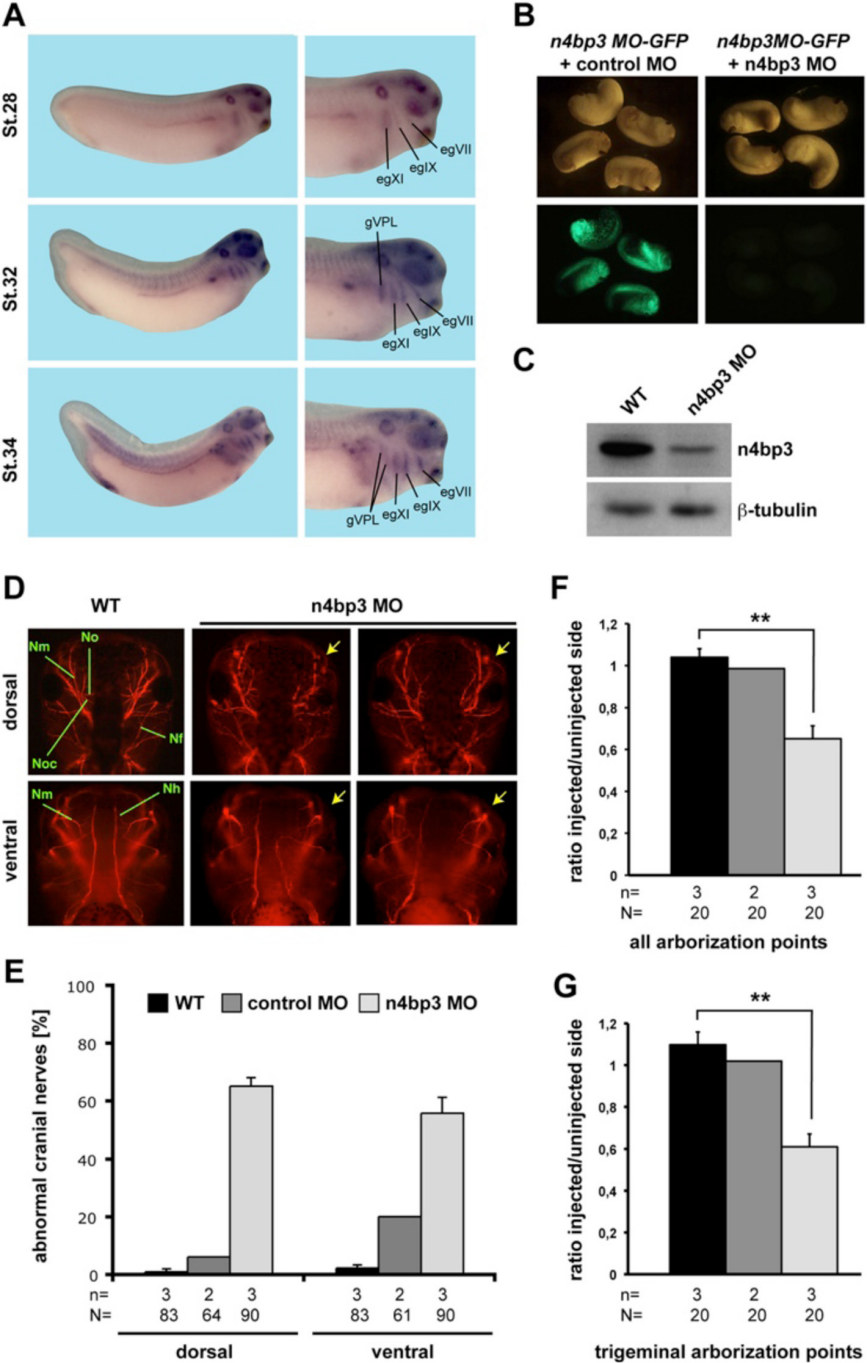
***In vivo *knockdown of n4bp3 in *Xenopus laevis *results in abnormal cranial nerve development. (A)** Spatiotemporal expression profile of *n4bp3* during early *X. laevis* embryogenesis. At stages 28, 32 and 34, *n4bp3* transcripts are detected in different cranial ganglia as indicated. **(B)** Coinjection of n4bp3 morpholino oligonucleotide green fluorescent protein (*n4bp3 MO-GFP*) RNA together with a control MO results in GFP expression, whereas coinjection with n4bp3 MO results in a block of GFP translation. **(C)** Western blot shows that n4bp3 protein level is strongly decreased upon n4bp3 MO injection compared to n4bp3 protein level in wild-type (WT) embryos. β-tubulin served as a loading control. **(D)** Unilateral injection of 20 to 25 ng of n4bp3 MO results in disturbed cranial nerve formation (yellow arrows), whereas control embryos (WT or control MO-injected) show normal cranial nerve development. **(E)** Quantitative presentation of the results shown in **(D)**. Statistical evaluation of all visible points of arborization of all cranial nerves **(F)** or the trigeminal nerve **(G)**. **(E)** through **(G)** Black bars, WT; dark gray bars, control MO; light gray bars, n4bp3 MO. For statistical evaluation in **(F)** and **(G)**, WT was compared with n4bp3 MO using Student’s *t*-test. **P < 0.01. egVII, facial epibranchial ganglion; egIX, glossopharyngeal epibranchial ganglion; egXI, first vagal epibranchial ganglion; gVPL, cells contributing to the vagal and posterior lateral line ganglion; n, independent experiments; N, number of investigated embryos; Nf, facial nerve; Nh, hypoglossal nerve; Nm, mandibular nerve; No, optical nerve; Noc, oculomotor nerve; st, stage.

On the basis of our localization studies, we cloned a functional N4BP3-interfering RNA (N4BP3-RNAi) construct (Figure 3A) and performed transient N4BP3-KD experiments in primary hippocampal neurons during the periods of axonal and dendritic development (DIV3 + 2, Figures 3B through 3G; DIV8 + 3, Figure 3H through 3J). To distinguish between axons and dendrites in these experiments, we immunostained all culture wells used for transfection with a phosphorylated inhibitor of κB, subunit α (phospho-IκBα) antibody, which clearly delineates the axon initial segment [26] (representative images are shown in Figure 3B). First, we found that loss of N4BP3 leads to a disruption of axonal arborization, whereas axonal length was not affected (Figure 3C and 3D). This phenotype was reflected quantitatively by a reduced number of (predominantly secondary and tertiary) branches per axon and by a decreased axon complexity index (ACI; see [27]) (Figure 3E through 3G). Second, we found that loss of N4BP3 not only impairs axonal development but also leads to disruption of dendritic branching (Figure 3H), as revealed by a reduced number of all dendritic end tips and by a downward shift of the corresponding Sholl plot (Figure 4I and 4J), when compared to empty control vector–transfected control neurons. To exclude off-target effects, we further generated a functional RNAi-resistant Myc-N4BP3 construct by site-directed mutagenesis (Additional file 1: Figure S1A). Cotransfection of primary hippocampal neurons with this construct and either the empty control vector or N4BP3 RNAi did not reveal any differences in the complexity of dendritic arbors either (Additional file 1: Figure S1B).

### *n4bp3* is essential for cranial nerve branching in developing *Xenopus laevis* embryos

To investigate *n4bp3* function *in vivo*, we made use of *X. laevis* as a model organism. First, we were interested in whether *n4bp3* is expressed in the developing nervous system of *X. laevis* and investigated its expression profile during early embryogenesis by whole-mount *in situ* hybridization (WMISH) experiments using a specific antisense n4bp3 RNA probe. We found that *n4bp3* transcripts are indeed detectable in developing brain structures, eyes, otic vesicles, heart, pronephros, liver and diverse cranial ganglia of *X. laevis* embryos (Figure 4A, Additional file 2: Figure S2). The specific expression of *n4bp3* in cranial nerves strongly argues for a role of n4bp3 during cranial ganglia development. To investigate this hypothesis, we designed an antisense MO based on the published *X. tropicalis* sequence [Xenbase:XM_002936850]. To test whether n4bp3 MO binds to its binding site, we cloned the n4bp3 MO binding site in front of and in frame with GFP (*n4bp3 MO-GFP*). Next, we injected the RNA of *n4bp3 MO-GFP*, together with either the control or n4bp3 MO. Coinjection of *n4bp3 MO-GFP* and the control MO led to GFP fluorescence, whereas embryos coinjected with *n4bp3 MO-GFP*, together with n4bp3 MO, showed no GFP fluorescence (Figure 4B). To test the specificity of n4bp3 MO, we injected the n4bp3 MO bilaterally into two-cell stage embryos, cultivated them until stage 15 and performed Western blot analysis to determine protein levels. Upon n4bp3 depletion, we found that n4bp3 protein level had strongly decreased compared to the wild type (WT) (Figure 4C).

Next, we injected n4bp3 MO into one animal dorsal blastomere of eight-cell stage *X. laevis* embryos to target anterior neural tissue, including developing cranial ganglia. As controls, we used either uninjected (WT) or control MO-injected embryos. At stage 46, we performed immunostaining experiments with the neurofilament-specific antibody 3A10 to detect cranial nerves using uninjected and MO-injected *X. laevis* embryos [28]. Unilateral loss of n4bp3 function resulted in abnormal cranial ganglia development, including shorter, and even absent, ganglia, as well as reduced cranial nerve arborization at the injected site (Figure 4D and 4E). In addition, significantly fewer arborization points were counted upon loss of n4bp3 (Figure 4F and 4G). The control MO-injected or uninjected embryos revealed no changes in cranial nerve formation. These *in vivo* data strongly support our findings in primary hippocampal cultures showing disturbed branching of axons and dendrites upon loss of N4BP3 function.

## Discussion

Ubiquitylation plays a decisive regulatory role during the establishment of neural polarity, neuritogenesis and synapse formation [11,12]. In this context, the ubiquitin ligase Nedd4 has emerged to be a crucial modulator. Previous studies have shown that Nedd4 is able to control axon arborization, dendrite branching and synaptic transmission [15,16,29,30]. However, its molecular interactions, its regulation and its functions in neurons are still far from being completely understood. We have therefore started to uncover the functional role of N4BP3 in the developing nervous system. This hitherto uncharacterized protein not only contains a central Nedd4-binding motif [22] but also exhibits a C-terminal Fez1 domain. This feature classifies N4BP3 as a member of the Fezzin family, a group of molecules that interacts with spine-associated Rap GTPase-activating proteins and the ProSAP/Shank platform in the postsynaptic density (PSD) of excitatory synapses via Fez1 and/or PDZ domain interaction, respectively [23-25]. However, N4BP3 exhibits the least conserved Fez1 domain among family members (see public databases such as NCBI) and contains only a rudimentary PDZ domain-binding motif. Therefore, N4BP3 might not exhibit its major functions within the PSD scaffold, as do other Fezzins. In fact, the presence of N4BP3 in neuronal processes, including axons and dendrites, implicates a role for this protein in early neuronal development. These observations are in line with the results of previous studies showing that N4BP3 interaction partner Nedd4 is also found in axons and growth cones of RGCs [15], as well as in neurites and growth cones of DRG sensory neurons [18]. N4PB3 thus not only binds Nedd4 [22] but also might have an impact on Nedd4-related processes in developing neurons. To test this hypothesis, we used an RNAi approach to transiently knock down N4PB3 and first conducted this experiment during the period of axonal branching of hippocampal neurons in culture [31]. The resulting phenotype, a loss of predominantly secondary and tertiary branches and a significantly decreased ACI, does in fact resemble the Nedd4-KD phenotype in axons [15,18]. Because Nedd4 is crucial not only for axonal branching but also for dendritic branching [16], we used the same approach used previously and transiently knocked down N4BP3 during the core period of dendritic branching in hippocampal cultures [32]. Our subsequent analyses demonstrate that loss of N4BP3 leads to a less complex dendritic arbor. These data resemble the phenotypical alterations of Nedd4 deficiency on dendrites as described previously [16]. Most interestingly, the Nedd4-binding motif in N4BP3 is conserved among species, including *X. laevis*. We reveal highly specific expression of *n4bp3* in the *X. laevis* nervous system, including cranial nerve ganglia. Strikingly, loss of n4bp3 via MO-mediated KD severely disrupts cranial nerve development in *X. laevis* embryos *in vivo*. These results provide strong support for previous work in Nedd4-deficient mice, which exhibit a failure in caliber, growth, fasciculation and axon number of the phrenic nerve early in development [19].

## Conclusions

On the basis of our loss of function studies *in vitro* and *in vivo*, we conclude that N4BP3 is essential for the proper development of neuronal processes among species. We further propose that N4BP3 could be crucial for Nedd4-related function during neural development. At this stage, however, two aspects are still unclear: (1) the molecular mechanism, that is, how N4BP3 might influence Nedd4 function; and 2) the consequences that a potential N4BP3-Nedd4 interplay in neurons have on Nedd4 substrates. In axons, for example, Nedd4 has repeatedly been shown to promote arborization via ubiquitylation of PTEN [15,18], a central inhibitor of PI3K signaling and cytoskeletal growth [33,34]. Moreover, Nedd4 is known to promote dendritic branching via ubiquitylation of Rap2 [16], a negative regulator of dendrite growth [35]. Based on the aforementioned facts, in future studies, it will be important to characterize a potential N4BP3-Nedd4 complex in neurons and analyze its impact on PTEN and Rap2 function during neurite development.

## Methods

### *In silico* analysis of the Nedd4-binding protein 3 domain structure

The domain structure of rat N4BP3 (coiled-coil and Fez1 domains) was analyzed online using the Pfam 26.0 database (Wellcome Trust Sanger Institute, Hinxton, UK).

### Cloning of Nedd4-binding protein 3

We found the rat cDNA sequence of the Fezzin family member N4BP3 in public database homology searches. Using a polymerase chain reaction (PCR)-based approach, we cloned a full-length N4BP3 cDNA using a rat total brain cDNA library (Stratagene, La Jolla, CA, USA) as a template. The correct cDNA was confirmed by DNA sequencing and inserted into different bacterial and eukaryotic expression vectors, including pEGFP and pCMV-myc (both from Clontech Laboratories, Mountain View, CA, USA).

### Antibodies and vector constructs

A partial cDNA of N4BP3 (encoding aa 1 to 309) was subcloned into the bacterial expression vector pGEX-4T (GE Healthcare, Freiburg, Germany). A glutathione *S*-transferase (GST)-N4BP3 fusion protein was expressed in *Escherichia coli* BL-21 and purified on Glutathione Sepharose 4B as recommended by the manufacturer (GE Healthcare). The purified fusion protein was used to generate a polyclonal N4BP3 antiserum in rabbits (Pineda Antibody Service, Berlin, Germany). This antiserum was subsequently affinity-purified using a HiTrap NHS-activated HP column loaded with GST-N4BP3 (GE Healthcare). The following primary antibodies were purchased from commercial suppliers: microtubule-associated protein 2 (MAP2; Aves Laboratories, Tigard, OR, USA), phospho-IκBα (Ser32/36) (Cell Signaling Technology, Danvers, MA, USA), GFP (Clontech Laboratories), neurofilament-associated antigen 3A10 (Developmental Studies Hybridoma Bank (DSHB), University of Iowa, Iowa City, IA, USA), Nedd4 (Novus Biologicals, Littleton, CO, USA), c-Myc (Roche Applied Science, Mannheim, Germany), β-tubulin (Sigma-Aldrich, Steinheim, Germany) and axonal growth cone antigen 2G13P (Thermo Scientific, Bonn, Germany).

The rat N4BP3- RNAi construct was generated following published methods using the pSUPER vector (Oligoengine, Seattle, WA, USA). We chose the target sequence for the N4BP3-RNAi (5′-AG ACT TGG CAG GAG GAA AA-3′) according to propositions made by Eurofins MWG Operon and the corresponding oligonucleotides were ordered from this company (Ebersberg, Germany). The RNAi-resistant Myc-N4BP3 construct was generated by site-directed mutagenesis using the QuikChange II XL Site-Directed Mutagenesis Kit (Stratagene) to introduce three different point mutations (5′-AG ACG TGG CAA GAG GAG AA-3′) within the RNAi target sequence.

### Biochemistry

For characterization of the affinity-purified N4BP3 antibody, recombinant Myc-N4BP3 protein (rat sequence) was generated using an *in vitro* TNT kit (Promega, Madison, WI, USA). Furthermore, HEK-293T cells (transfected with GFP-N4BP3 or Myc-N4BP3, as well as untransfected cells) or primary hippocampal cultures were lysed in Triton X-100 lysis buffer (150 mM NaCl, 50 mM Tris•HCl, 1% Triton X-100, pH 8.0, protease inhibitor mix; Roche Applied Science). Protein concentrations were determined by Bradford protein assay, and 15 μg of protein per lane were loaded for SDS-PAGE. Western blot analysis was conducted following standard protocols. Horseradish peroxidase-conjugated secondary antibodies (Dako, Glostrup, Denmark) and the SuperSignal Detection System (Thermo Scientific) were used to visualize protein bands on X-ray films (GE Healthcare).

### Culturing of rat primary hippocampal neurons

The preparation of hippocampal cultures from rat was performed at embryonic stage 18 (E18), essentially as described previously [36]. In brief, hippocampal neurons were seeded onto poly-L-lysine (0.1 mg/ml; Sigma-Aldrich) coated glass coverslips. Cells were grown in Neurobasal Medium complemented with B-27 supplement, 0.5 mM L-glutamine and penicillin/streptomycin at 100 U/ml (all reagents were purchased from Life Technologies, Darmstadt, Germany) and maintained at 37°C in a 5% CO_2_ atmosphere.

### Immunocytochemistry

Cultured cells were fixed with 4% paraformaldehyde (PFA)/1.5% sucrose in phosphate-buffered saline (PBS) at room temperature for 20 min and processed for immunocytochemistry. After permeabilization of the cells with 0.1% Triton X-100 in PBS for 5 min, blocking was performed using 5% fetal calf serum in PBS followed by the primary antibody at 4°C overnight. Washing with PBS was followed by incubation with the secondary antibody coupled to Alexa Fluor 488, 568 or 647 (all from Molecular Probes) for 1 h at room temperature. The actin cytoskeleton was visualized using Alexa Fluor 647 phalloidin in some experiments. Cell nuclei were counterstained with 4′,6-diamidino-2-phenylindole, and, after further washing steps, cells were mounted in Mowiol medium. Images were captured using an upright fluorescence microscope (Axioskop 2; Zeiss, Oberkochen, Germany). For Sholl analysis, concentric circles (15, 30, 45, 60, 75, 90, 105 and 120 μm in diameter) were drawn around the soma of each neuron included in the analysis. The number of all dendrites crossing each circle was counted manually.

### Transfections

Vector constructs were transfected into HEK-293T or COS-7 cells using PolyFect reagent (QIAGEN, Hilden, Germany) or into hippocampal neurons using Lipofectamine 2000 reagent (Life Technologies).

### *Xenopus laevis* embryos

*X. laevis* embryos were generated by *in vitro* fertilization, cultured in 0.1× modified Barth’s saline solution buffer (88 mM NaCl, 1 mM KCl, 2.4 mM NaHCO_3_, 0.82 mM MgSO_4_•7H_2_O, 0.33 mM Ca(NO_3_)_2_•4H_2_O, 0.41 mM CaCl•6H_2_O, 10 mM HEPES, pH 7.6) and staged according to a previously described protocol [37]. Embryos were fixed either with MEMFA (0.1 M 3-(N-morpholino)propanesulfonic acid (pH 7.4), 2 mM ethylene glycol tetraacetic acid, 1 mM MgSO_4_ and 4% formaldehyde) for WMISH experiments or with 4% PFA in PBS for antibody staining. For later Western blot analysis, embryos were frozen at −80°C.

### Whole-mount *in situ* hybridization and immunostaining in *Xenopus laevis*

To visualize the spatiotemporal expression pattern of *n4bp3* during *X. laevis* embryogenesis, WMISH experiments were performed using a 1.447-kb digoxigenin-labeled antisense *n4bp3* RNA probe detecting *X. laevis n4bp3* mRNA. For cloning of the *n4bp3* RNA probe, we used the following primers based on the published *X. tropicalis* sequence [Xenbase:XM_002936850]: n4bp3_l: 5′-ATG GGA AGT GTA AGC AGC CTA-3′; and n4bp3_r: 5′-TCT GGG CTT CAT TCT GAC GC-3′. PCR was performed using Phusion High-Fidelity DNA Polymerase (Thermo Fisher Scientific, Pittsburgh, PA, USA) and *X. laevis* cDNA isolated from stage 20 embryos. The PCR product was cloned into the pSC-B vector (Stratagene), and the antisense RNA probe was generated using NotI enzyme (New England Biolabs, Ipswich, MA, USA) and T3 RNA Polymerase (Roche Applied Science). WMISH experiments were performed using *X. laevis* embryos at different developmental stages according to standard protocols [38]. For a more detailed analysis of *n4bp3* expression, we studied vibratome sections [39]. The monoclonal 3A10 antibody (DSHB) was further used to visualize cranial nerve fibers at E46 by immunohistochemical staining according to the method outlined by Schuff *et al*. [28].

### Microinjection in *Xenopus laevis* embryos

For loss of function experiments in *X. laevis* embryos, antisense MOs were obtained from Gene Tools (Philomath, OR, USA), resuspended in diethylpyrocarbonate-treated H_2_O and stored in aliquots at −20°C. The standard control MO sequence is 5′-CCT CTT ACC TCA GTT ACA ATT TAT A-3′, and the sequence of the n4bp3 MO is 5′-CAA TCA GGC TGC TTA CAC TTC CCA T-3′. Twenty to twenty-five nanograms of either MO were injected unilaterally in one animal dorsal blastomere of eight-cell stage embryos targeting anterior neural tissue. Correct injections were controlled by the coinjection of *GFP* RNA (Additional file 3: Figure S3), which was generated by *in vitro* transcription using the mMESSAGE mMACHINE Kit (Life Technologies). To test the translational blocking efficiency of n4bp3 MO, the n4bp3 MO binding site was cloned in front of and in frame with *GFP* in pCS2+ vector (*n4bp3 MO-GFP*). One nanogram of *n4bp3 MO-GFP* RNA was then injected bilaterally into *X. laevis* embryos at two-cell stage, together with 25 ng of either the standard control or n4bp3 MO. GFP fluorescence was monitored at stage 24 of development. Furthermore, n4bp3 MO was injected bilaterally into two-cell stage *X.* embryos. At stage 15, embryos were fixed. Protein lysates were generated as described previously [40] and subsequently analyzed by Western blotting.

### Statistical analysis

For cell culture experiments, ten cells from three independent experiments per condition were analyzed using AxioVision version 4.8.2 software (Zeiss). For the statistical analysis of cranial nerve branching in *X. laevis* embryos, 20 embryos (WT, n4bp3 MO) and 23 control MO embryos from three experiments (WT, n4bp3 MO) and two experiments (control MO), respectively, were examined. All data were tested for significance by using Student’s *t*-test (*P < 0.05, **P < 0.01, ***P < 0.001). All animal experiments in this study were performed in accordance with the guidelines for the welfare of experimental animals issued by the federal government of Germany and by the local ethics committee at Ulm University (ID 0.103).

## Competing interests

The authors declare that they have no competing interests.

## Authors’ contributions

MJS, SJK, MK and TMB conceived the outline of this study. MJS performed all biochemistry and cell culture experiments together with TMW, MS and AMG. SJK and NHB performed all experiments in *Xenopus laevis* embryos. All authors analyzed the data and jointly wrote the manuscript. All authors read and approved the final manuscript.

## Supplementary Material

Additional file 1: Figure S1 Specificity testing of the Nedd4-binding protein 3 interfering RNA construct. **(A)** Characterization of the interfering RNA (RNAi)-resistant Myc-Nedd4-binding protein 3 (Myc-N4BP3) construct. HEK-293T cells were cotransfected with Myc-N4BP3 (left panel) or Myc-N4BP3 RNAi-res (right panel) and either the empty pSUPER control vector (Oligoengine, Seattle, WA, USA) or the N4BP3 RNAi construct, respectively. Western blot of the corresponding cell lysates shows a clear reduction of Myc-N4BP3 and almost no reduction of Myc-N4BP3 RNAi-res in the presence of N4BP3 RNAi. Myc antibodies were used to detect Myc-N4BP3 or Myc-N4BP3 RNAi-res (marked by asterisk). β-actin was used as a loading control. **(B)** Representative images (left panel) of rat hippocampal neurons transiently cotransfected (DIV8 + 3) with Myc-N4BP3 RNAi-res and either the empty pSUPER control vector (black squares) or the N4BP3 RNAi construct (gray squares), as indicated. Corresponding Sholl analysis (right panel). No significant differences were observed (*n* = 10 cells from three independent experiments). Click here for file

Additional file 2: Figure S2 Expression pattern of *n4bp3* in Xenopus laevis. **(A)** During gastrulation, *n4bp3* is expressed in the mesoderm surrounding the blastoporus (vegetal view). **(B)** and **(C)** Anterior views (dorsal to the top). **(D)** and **(I)** Ventral views anterior to the right (D) or anterior to the top (I). **(E)** through **(H)** Lateral views with anterior to the right. Dashed lines show levels of sections as indicated. Dashed circles indicate the cement gland. **(J)** through **(N)**, **(P)**, **(R)** and **(S**) Transverse sections. **(O)** and **(Q)** Horizontal sections. *n4bp3* is expressed in the otic vesicles (black arrowheads), the neural tube (red arrows), the pronephros (black arrows), the liver (white arrows), the heart (white arrowheads), the foregut endoderm (green arrow) and the developing eye (green arrowheads). egVII, facial epibranchial ganglion; egIX, glossopharyngeal epibranchial ganglion; egXI, first vagal epibranchial ganglion; gVPL, cells contributing to the vagal and posterior lateral line ganglion. Click here for file

Additional file 3: Figure S3 Controlling morpholino injections in Xenopus laevis embryos. For all morpholino oligonucleotide injection experiments, *GFP* RNA was coinjected to collect for correctly injected embryos. Green fluorescent protein (GFP) was checked under the fluorescence microscope between stages 20 and 26. Only embryos that showed strong unilateral glowing in the head region were used for further experiments. Click here for file
