# Supplementary figures and images for: The Nedd4-binding protein 3 (N4BP3) is crucial for axonal and dendritic branching in developing neurons
Source: Neural Dev. 2013 Sep 17;8:18. doi: 10.1186/1749-8104-8-18 (PMC3849298; doi:10.1186/1749-8104-8-18)

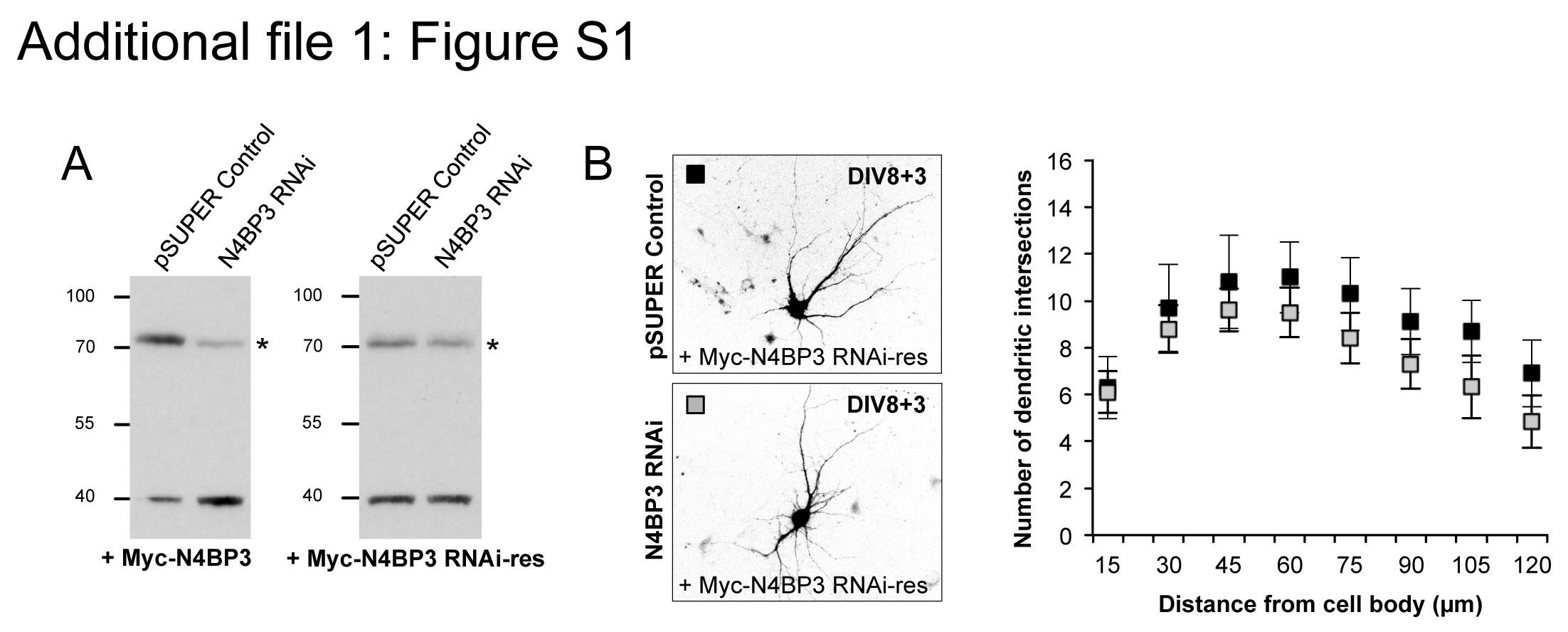

Supplement: Additional file 1: Figure S1 — Specificity testing of the Nedd4-binding protein 3 interfering RNA construct. (A) Characterization of the interfering RNA (RNAi)-resistant Myc-Nedd4-binding protein 3 (Myc-N4BP3) construct. HEK-293T cells were cotransfected with Myc-N4BP3 (left panel) or Myc-N4BP3 RNAi-res (right panel) and either the empty pSUPER control vector (Oligoengine, Seattle, WA, USA) or the N4BP3 RNAi construct, respectively. Western blot of the corresponding cell lysates shows a clear reduction of Myc-N4BP3 and almost no reduction of Myc-N4BP3 RNAi-res in the presence of N4BP3 RNAi. Myc antibodies were used to detect Myc-N4BP3 or Myc-N4BP3 RNAi-res (marked by asterisk). β-actin was used as a loading control. (B) Representative images (left panel) of rat hippocampal neurons transiently cotransfected (DIV8 + 3) with Myc-N4BP3 RNAi-res and either the empty pSUPER control vector (black squares) or the N4BP3 RNAi construct (gray squares), as indicated. Corresponding Sholl analysis (right panel). No significant differences were observed (n = 10 cells from three independent experiments). [file 1749-8104-8-18-S1.tiff]

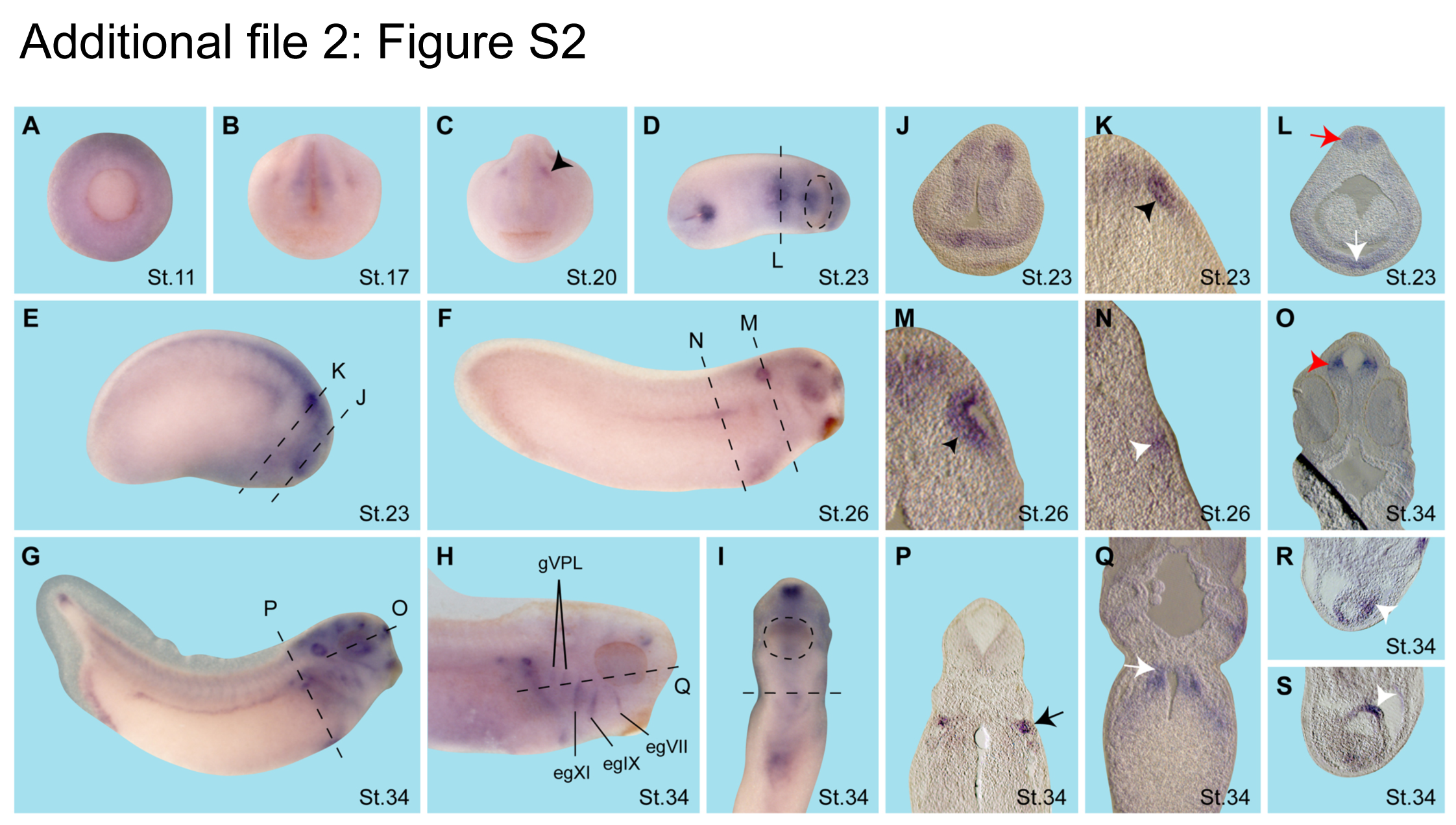

Supplement: Additional file 2: Figure S2 — Expression pattern of n4bp3 in Xenopus laevis. (A) During gastrulation, n4bp3 is expressed in the mesoderm surrounding the blastoporus (vegetal view). (B) and (C) Anterior views (dorsal to the top). (D) and (I) Ventral views anterior to the right (D) or anterior to the top (I). (E) through (H) Lateral views with anterior to the right. Dashed lines show levels of sections as indicated. Dashed circles indicate the cement gland. (J) through (N), (P), (R) and (S) Transverse sections. (O) and (Q) Horizontal sections. n4bp3 is expressed in the otic vesicles (black arrowheads), the neural tube (red arrows), the pronephros (black arrows), the liver (white arrows), the heart (white arrowheads), the foregut endoderm (green arrow) and the developing eye (green arrowheads). egVII, facial epibranchial ganglion; egIX, glossopharyngeal epibranchial ganglion; egXI, first vagal epibranchial ganglion; gVPL, cells contributing to the vagal and posterior lateral line ganglion. [file 1749-8104-8-18-S2.tiff]

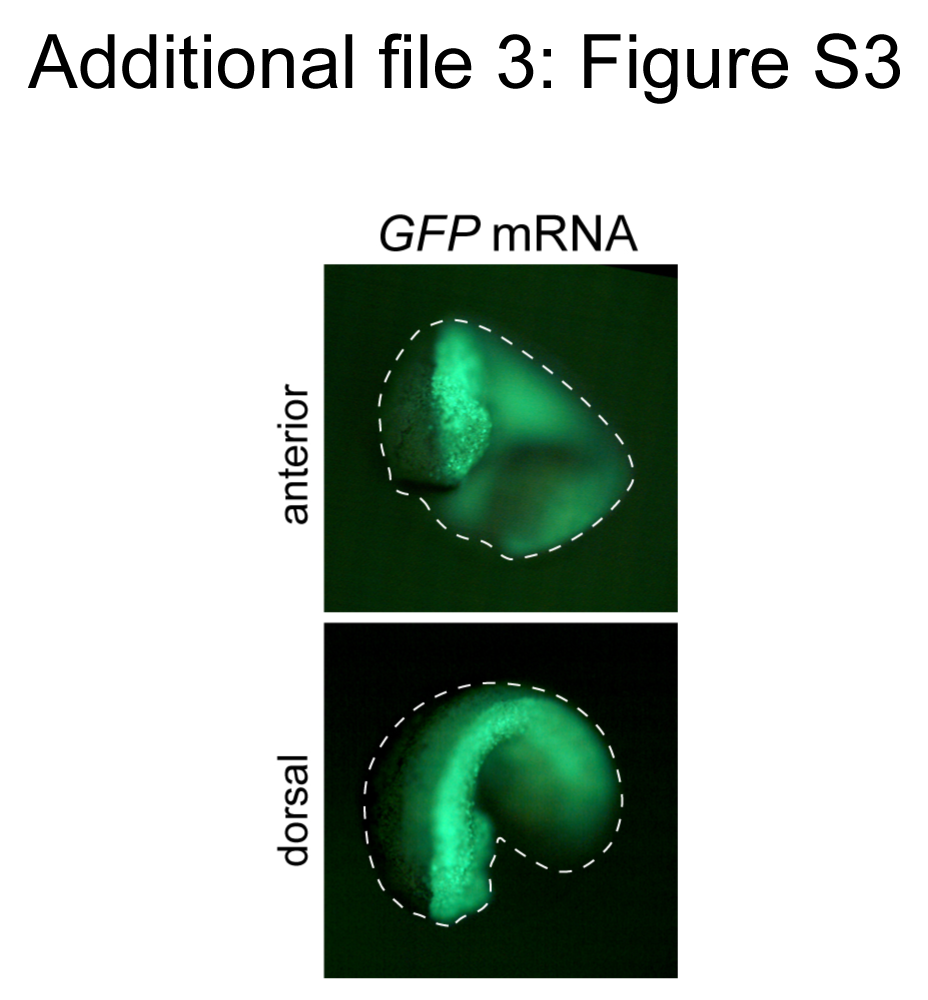

Supplement: Additional file 3: Figure S3 — Controlling morpholino injections in Xenopus laevis embryos. For all morpholino oligonucleotide injection experiments, GFP RNA was coinjected to collect for correctly injected embryos. Green fluorescent protein (GFP) was checked under the fluorescence microscope between stages 20 and 26. Only embryos that showed strong unilateral glowing in the head region were used for further experiments. [file 1749-8104-8-18-S3.tiff]
